# Predictive factors of infection in patients with chronic kidney disease using hemodialysis catheters

**DOI:** 10.1590/1677-5449.202200982

**Published:** 2023-08-11

**Authors:** Juliana da Costa Matos, Laura Lane Menezes Polsin, Karla Cristina Petrucelli Israel, Leonardo Pessoa Cavalcante

**Affiliations:** 1 Universidade Federal do Amazonas - UFAM, Manaus, AM, Brasil.; 2 Universidade do Estado do Amazonas - UEA, Manaus, AM, Brasil.

**Keywords:** renal dialysis, hemodialysis units, hospital, catheter-related infections, central venous catheters

## Abstract

**Background:**

Infection is the most frequent complication of central venous catheters used for hemodialysis.

**Objectives:**

The purpose of this study was to the determine the central venous catheter-related infection rate at a dialysis center in the Brazilian state of Amazonas and to identify risk factors and the microbiological profile of the infections.

**Methods:**

This was an observational study with prospective data collection over a 12-month period by chart analysis and face-to-face interviews with patients undergoing hemodialysis using central venous catheters at a dialysis center.

**Results:**

96 central venous catheters were analyzed in 48 patients. 78 of these were non-tunneled central venous catheters (81.3%) and 18 were tunneled central venous catheters (18.7%), 53.1% of the catheters were exchanged because of infection and blood cultures were obtained from 35.2% of the patients who had catheter-related infections. Gram-negative bacteria were isolated from five of the nine blood cultures in which there was bacterial growth and Gram-positive bacteria were isolated from the other four. The most commonly isolated bacteria was *Staphylococcus hominis*, found in 22.2% of positive blood cultures.

**Conclusion:**

The overall hemodialysis venous catheter infection rate was 10.1 episodes/1000 catheter days, 15.1 episodes/1000 catheters days in non-tunneled catheters and 3.3 episodes/1000 catheters days in tunneled catheters. The infection predisposing factors identified were use of non-tunneled catheters and having 2 hemodialysis sessions per week. Regarding the microbiological profile, over half of the bacteria isolated were Gram-negative.

## INTRODUCTION

Hemodialysis treatment is administered via long or short term vascular access. Short-term accesses employ non-tunneled (without a subcutaneous cuff) central venous catheters (CVCs), while long-term accesses should use a tunneled CVC (with a subcutaneous cuff) or an arteriovenous fistula (AVF) should be used as the definitive hemodialysis access. CVCs are the most often used type of initial hemodialysis vascular access, even in patients who were being treated by a nephrologist for up to 4 months before starting renal replacement therapy (RRT).^1^ Infection is the most frequent complication related to use of CVCs for hemodialysis, and the mere presence of a CVC is the principal risk factor for bacteremia/infection.^2^ Astor et al.^3^ calculated a 47% greater risk of mortality in patients using CVCs for hemodialysis, compared with patients receiving dialysis via an AVF.

CVC infections manifest clinically with fever and/or shivering, in addition to possible local signs of infection, such as hyperemia and/or secretion at the site of catheter insertion, or nearby.^4^ According to the Infectious Disease Society of America,^5^ etiologic diagnosis of catheter-related infection is confirmed when the same microorganism grows in a peripheral blood sample and a catheter tip sample or in two different peripheral blood samples.

In view of the above, this study aimed to determine the rate of incidence of hemodialysis CVC-related infection and possible predictive factors and also to find out the microbiological profile of these infections at a dialysis center in the Brazilian state of Amazonas.

## METHODS

This is a prospective, observational study of incidence, approved by the Research Ethics Committee at the Universidade Federal do Amazonas (decision number 4.231.319) and conducted from October 2020 to October 2021 at the Amazonas Renal Diseases Center, a private clinic specialized in treating patients with kidney disease that is accredited to the Public Unified Health System (SUS - Sistema Único de Saúde) and has an average of 240 patients on its hemodialysis program.

**Figure 1:**
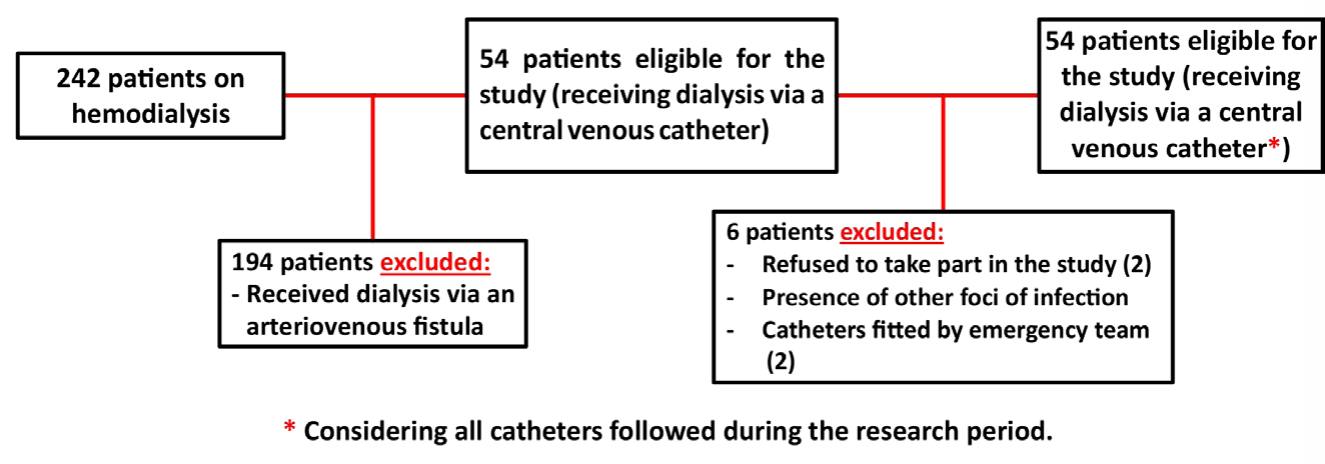
Flowchart illustrating eligibility for enrollment in central venous catheter sample at the Amazonas Renal Diseases Center.

The sample comprised patients with chronic kidney disease (CKD) who were on a hemodialysis program receiving three hemodialysis sessions per week, with a mean duration of 3 and a half hours, and were using CVCs. The inclusion criteria were: 1) chronic renal patients; and 2) receiving dialysis via a CVC implanted at the dialysis center. The exclusion criterion was the presence of another infectious site. The sample size calculation considered 54 patients on dialysis via central venous catheters (Figure 1), a 95% confidence level, a 5% sampling error, and an estimated 55% frequency of bacteremia in central venous catheters,^6,7^ resulting in a minimum of 48 patients considered necessary for the sample. The sample size calculation was performed using WinPEPI.^8^

Data were collected in face-to-face interviews with patients conducted during their hemodialysis sessions and additional information was retrieved from their medical records. A structured chart was used for demographic data, laboratory test results, comorbidities, details of each patient’s current RRT, serology, prior history of interventions and hospitalizations, time since onset of CKD, signs and symptoms, diagnoses of systemic infection and/or infection at the catheter insertion site, blood culture results, empirical treatment given, and outcome of infection.

Data were stored in an Excel spreadsheet and analyzed with the Statistical Package for the Social Sciences - SPSS, version 21.0. The normality of the distribution of quantitative data was verified using the Kolmogorov Smirnov test and variables with normal distribution were expressed as mean and standard deviation and those with asymmetrical distribution as median and interquartile range. Categorical variables were presented as absolute and relative values. Comparisons between quantitative variables were evaluated using Student’s *t* test and the Mann-Whitney test and associations between categorical variables were analyzed with Pearson’s chi-square test and Fischer’s exact test. Results with p < 0.05 were considered statistically significant.

## RESULTS

A total of 48 patients (Figure 1) were enrolled, with a mean age of 54.3±14.4 years, ranging from 24 to 83 years (p = 0.909), with majorities of females (52.1%) (p =0.554), patients with diabetes mellitus (DM) (60.4%) (p = 0.420), and patients with systemic arterial hypertension (SAH) (89.4%) (p = 0.504). The most frequent body weight categories were normal weight (37.0%) and overweight (39.1%) (p = 0.565). Majorities of the patients received dialysis three times per week (91.7%) (p = 0.009), had not been admitted to hospital during the previous 30 days (74.2%) (p = 0.490), and had not had transplants (94.3%) (p = 0.369). There were five transplanted patients, two had had transplants 10 years previously and another two had had transplants 21 years previously (Table 1) (p = 0.999).

**Table 1:** Infections associated with sociodemographic, anthropometric, clinical, and health characteristics of patients with chronic kidney disease on a hemodialysis program at the Amazonas Renal Diseases Center, considering all catheters (n = 96).

**Characteristics**	**Infection**		**p**
	**No**	**Yes**	
	**n = 42 n (%)**	**n = 54 n (%)**	
Sociodemographic			
Age in years (mean±SD)	54.7±14.2	55.1±12.9	0.909^a^
Sex			0.554^b^
Male	20 (47.6)	29 (53.7)	
Female	22 (54.4)	25 (46.3)	
Anthropometric			
Weight in kg (mean±SD)	68.8±12.9	67.2±11.4	0.527^a^
Height in m (mean±SD)	1.6±0.1	1.6±0.1	0.246^a^
BMI in kg/m^2^ (mean±SD)	25.7±4.6	25.8±3.8	0.881^a^
Nutritional status			0.565^c^
Underweight (< 18.5)	4 (9.8)	2 (3.9)	
Healthy weight (18.5-24.9)	14 (34.1)	24 (47.1)	
Overweight (25-29.9)	11 (26.8)	13 (25.5)	
Obesity (30-39.9)	12 (29.3)	12 (23.5)	
Laboratory analyses			
Hemoglobin g/dL (mean±SD)	9.6±1.8	9.4±1.4	0.467^a^
Albumin g/L (mean±SD)	3.7±05	4.0±1.4	0.233^a^
Iron µg/dL (median and IR)	49 (38-70)	57 (43-78)	0.279^d^
Ferritin ng/mL (median and IR)	228 (124-724)	500 (192-670)	0.306^d^
Preexisting diseases			
Diabetes mellitus			0.420^b^
No	16 (38.1)	25 (46.3)	
Yes	26 (61.9)	29 (53.7)	
Time since diagnosis of DM in years	20 (13-22)	17 (15-23)	0.943^d^
Systemic arterial hypertension			0.504^c^
No	3 (7.1)	7 (13.2)	
Yes	39 (92.9)	46 (86.8)	
Time since diagnosis of SAH in years (median and IR)	7 (3-10)	7 (3-18)	0.902^d^
Weekly dialysis frequency			0.009^a^
Twice	0 (0.0)	8 (14.8)	
Three times	42 (100.0)	46 (85.2)	
Hospital admission in last 30 days			0.490^b^
No	22 (78.6)	27 (71.1)	
Yes	6 (21.4)	11 (28.9)	
Transplant patient			0.369^a^
No	39 (97.5)	43 (91.5)	
Yes	1 (2.5)	4 (8.5)	
Years since transplant			0.999^a^
10	1 (100.0)	1 (25.0)	
13	0 (0.0)	1 (25.0)	
21	0 (0.0)	2 (50.0)	

a Student’s *t* test.

b Pearson’s chi-square test.

c Fischer’s exact test.

d Mann-Whitney test.

SD = standard deviation; DM = diabetes mellitus; SAH = systemic arterial hypertension; BMI = body mass index; IR = Interquartile range. Note: there was 1 missing value for each of systemic arterial hypertension, height, body mass index, and nutritional status; 15 missing values for time since diagnosis of diabetes mellitus; 20 for hemoglobin; 32 for albumin and iron; 35 for ferritin; and 43 for time since diagnosis of systemic arterial hypertension.

**Figure 2:**
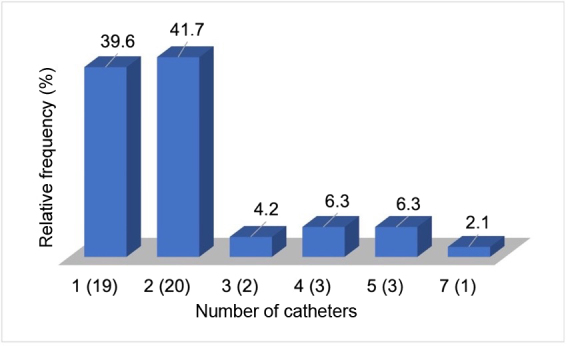
Frequency of patients with chronic kidney disease on a hemodialysis program at the Amazonas Renal Diseases Center, according to the number of catheter changes (n = 48).

Patients were assessed during consultations at the Amazonas Renal Diseases Center and the distribution of patients by number of catheter changes is illustrated in Figure 2. The majority of patients had one (39.6%) or two catheter changes (41.7%). The performance of a total of 96 catheters was monitored over the course of the study period and, considering the total number of assessments, the majority of patients were using non-tunneled catheters at study outset (81.7%) (p = 0.270) and had the same type of catheter implanted when they were exchanged during the study (81.3%) (p = 0.100). The most frequent access site was the right internal jugular vein (33.3%) (p = 0.382) and the majority of catheter changes were because of infection (53.1%) (p = 0.011). Most patients had negative serology (94.8%) (p = 0.129) and were positive for bacteremia (64.9%) (p = 0.388) with a median of two bacteremia episodes over the follow-up period (p = 0.120). When catheters were exchanged, the majority needed to change access site (73.4%) (p = 0.936) (Table 2).

**Table 2:** Frequencies of variables related to dialysis, accesses, and analysis and identification of access infections in patients with chronic kidney disease on a hemodialysis program at the Amazonas Renal Diseases Center, considering all catheters (n = 96).

**Variables**	**n (%)**
Access in use before study outset	
Tunneled catheter	5 (5.4)
Non-tunneled catheter	76 (81.7)
Arteriovenous fistula	7 (7.5)
None	5 (5.4)
Access fitted during study	
Tunneled catheter	18 (18.8)
Non-tunneled catheter	78 (81.3)
Access site	
Right femoral	19 (19.8)
Left femoral	16 (16.7)
Right jugular	32 (33.3)
Left jugular	21 (21.9)
Right subclavian vein	4 (4.2)
Left subclavian vein	4 (4.2)
Reason for changing access during study	
Infection	51 (53.1)
Tunneled access	10 (12.0)
Bleeding	2 (2.4)
No flow	16 (19.3)
Thrombosis	4 (4.8)
Serology	
Negative	91 (94.8)
Hepatitis B	1 (1.0)
Hepatitis C	4 (4.2)
Bacteremia	
No	26 (35.1)
Yes	48 (64.9)
Number of episodes of bacteremia (median and IR)	2 (1-3)
Admission during previous 30 days	
No	49 (74.2)
Yes	17 (25.8)
Previous change of access site	
No	25 (26.6)
Yes	69 (73.4)
Guidewire used to insert new catheter	
No	24 (25.5)
Yes	70 (72.9)
Blood culture	
No	75 (78.9)
Yes	20 (21.1)
Number of samples for blood culture	
One	1 (11.1)
Two	8 (88.9)
Samples not collected from the catheter tip	95 (100.0)
Number of days free from infection (median and IR)	39.5 (17.0-75.0)

IR = interquartile range. Note: there was 1 missing value for blood culture; 2 for previous change of access site and guidewire used to insert new catheter; 3 for access in use before study outset; 13 for reason for changing access during study; 22 for bacteremia; and 30 for admission during previous 30 days.

**Figure 3:**
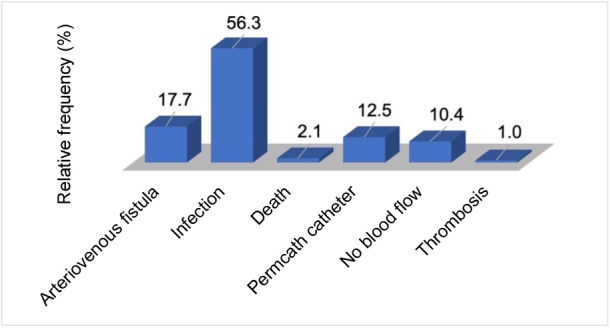
Frequency of outcomes, for all catheters, related to dialysis access of patients with chronic kidney disease on a hemodialysis program at the Amazonas Renal Diseases Center (n = 96).

Fifty-four of the 96 catheters evaluated developed infections during the study period (56.3%), which was the most common of all of the outcomes analyzed (Figure 3). Overall, 80% of the non-tunneled CVCs became infected during the study period and just 13% of the tunneled CVCs became infected over the same period (p = 0.100). Only 17.7% of the CVCs were removed due to a matured AVF. Patients whose catheters became infected were more likely to be receiving dialysis twice a week, whereas those who did not have infections were having three dialysis sessions per week (p = 0.009). No statistically significant differences were observed regarding any of the other characteristics analyzed in terms of presence or absence of infections (Table 1).

Patients with infections had catheters exchanged because of thrombosis more often, whereas patients without infections had their access converted to a tunneled catheter (p = 0.011). Blood cultures were ordered for patients with infections, but not for those without infections (p < 0.001) (Table 3).

**Table 3:** Infection associated with variables related to dialysis, to access, and to analysis and identification of access infection in patients with chronic kidney disease on a hemodialysis program at the Amazonas Renal Diseases Center, considering all catheters (n = 96).

**Characteristics**	**Infection**		**p**	
	**No**	**Yes**		
	**n = 42 n (%)**	**n = 54 n (%)**		
Access in use before study outset			0.270^a^	
Tunneled catheter	1 (2.4)	4 (7.8)		
Non-tunneled catheter	38 (90.5)	38 (74.5)		
Arteriovenous fistula	2 (4.8)	5 (9.8)		
None	1 (2.4)	4 (7.8)		
Access fitted during study			0.100^b^	
Tunneled catheter	11 (26.2)	7 (13.0)		
Non-tunneled catheter	31 (73.8)	47 (87.0)		
Access site			0.382^a^	
Right femoral	7 (16.7)	12 (22.2)		
Left femoral	10 (23.8)	6 (11.1)		
Right jugular	13 (31.0)	19 (35.2)		
Left jugular	7 (16.7)	14 (25.9)		
Right subclavian vein	2 (4.8)	2 (3.7)		
Left subclavian vein	3 (7.1)	1 (1.9)		
Reason for changing access during study:			0.011^a^	
Infection	22 (52.4)	29 (53.7)		
Tunneled access	9 (21.4)	1 (1.9)		
Bleeding	1 (2.4)	1 (1.9)		
No flow	8 (19.0)	8 (14.8)		
Thrombosis	0 (0.0)	4 (7.4)		
Reason for change noted in medical record and unknown to patient	2 (4.8)	11 (20.4)		
Serology			0.129^a^
Negative	42 (100.0)	49 (90.7)	
Hepatitis B	0 (0.0)	1 (1.9)	
Hepatitis C	0 (0.0)	4 (7.4)	
Bacteremia			0.388^b^
No	13 (40.6)	13 (31.0)	
Yes	19 (59.4)	29 (69.0)	
Number of episodes of bacteremia (median and IR)	2.5 (2.0-3.0)	1.5 (1.0-3.0)	0.120^c^
Previous change of access site			0.936^b^
No	11 (26.2)	14 (26.9)	
Yes	31 (73.8)	38 (73.1)	
Guidewire used to insert new catheter			0.895^b^
No	11 (26.2)	13 (25.0)	
Yes	31 (73.8)	39 (75.0)	
Blood culture			< 0.001^b^
No	40 (97.6)	35 (64.8)	
Yes	1 (2.4)	19 (35.2)	
Number of samples for blood culture			0.999^a^
One	0 (0.0)	1 (12.5)	
Two	1 (100.0)	7 (87.5)	
Number of days free from infection (median and IR)	39 (19-70)	40 (16-85)	0.960^c^

a Fischer’s exact test.

b Pearson’s chi-square test.

c Mann-Whitney test.

IR = interquartile range.

Note: there was 1 missing value for blood culture; 2 for previous change of access site and guidewire used to insert new catheter; 3 for access in use before study outset; 22 for bacteremia; and 30 for admission during previous 30 days.

**Figure 4:**
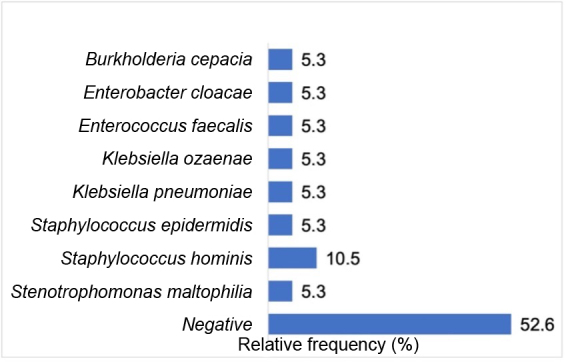
Frequency of results of blood cultures for all catheters in patients with chronic kidney disease on a hemodialysis program at the Amazonas Renal Diseases Center (n = 19).

Blood cultures were ordered for 19 (35.2%) (p < 0.01) patients who exhibited catheter infection, most often with two samples (88.9%) (p = 0.999). There was bacterial growth in nine patients’ cultures (47.4%). In five of these positive cultures the bacteria were gram-negative and in four the bacteria were gram-positive. Although more cultures were positive for gram-negative bacteria, the most frequently isolated bacteria was *Staphylococcus hominis,* which is gram-positive and was found in two of the nine positive blood cultures (Figure 4).

**Figure 5:**
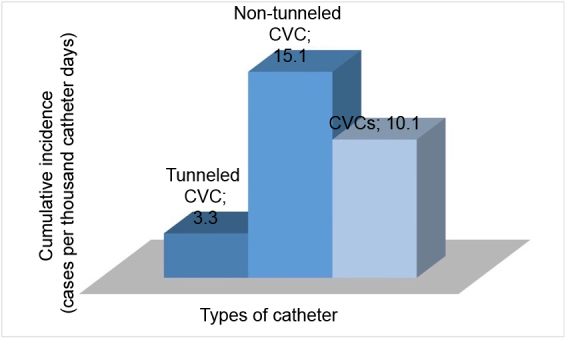
Cumulative incidence of infection, according to type of catheter, in patients with chronic kidney disease on a hemodialysis program at the Amazonas Renal Diseases Center (n = 96). CVC = central venous catheter.

The incidence of infection was higher in non-tunneled catheters than in tunneled ones (Figure 5). The overall median number of days free from infection was 39.5 (17.0-75.0) (Table 2).

**Figure 6:**
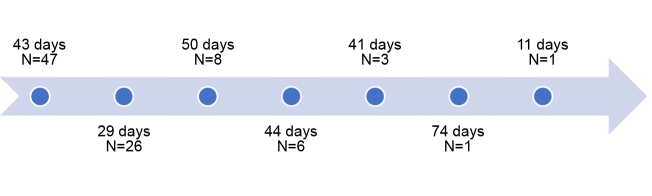
Timeline of median days free from infection between each catheter change in patients with chronic kidney disease on a hemodialysis program at the Amazonas Renal Diseases Center.

Figure 6 shows a timeline illustrating the median number of days free from infection from baseline to the first catheter change, and so on. The N indicates how many patients had each number of catheter changes.

**Figure 7:**
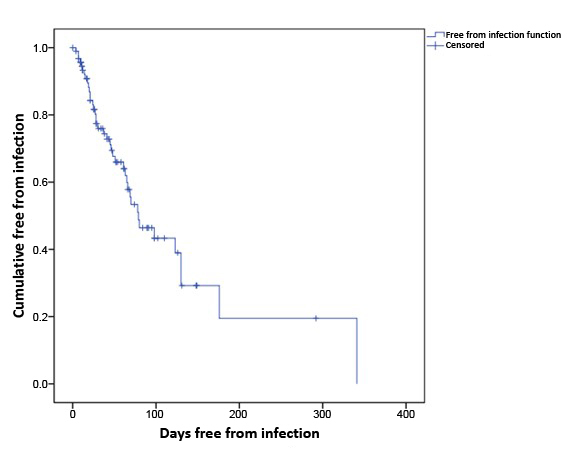
Kaplan-Meier curve of days free from infection in patients with chronic kidney disease on a hemodialysis program at the Amazonas Renal Diseases Center (n = 94).

The median number of days without infection was 79 days (95% confidence interval [95%CI] 48-110) (p = 0.960). For the whole sample, 98.9% of patients remained free from infection for 4 days, 51.1% remained infection free for 78 days, and 19.5% remained 176 days without an infection (Figure 7).

## DISCUSSION

Prevention and control of infections is a constant concern among healthcare professionals caring for chronic renal patients on CVC hemodialysis programs. Alhazmi et al.,^6^ conducted a retrospective study in Saudi Arabia analyzing 160 patients and correlated use of CVCs as an independent risk factor for infection. Type of vascular access is a recognized risk factor for bloodstream infection in hemodialysis patients and AVFs are the ideal type of vascular access because they are associated with lower rates of infectious complications.^9^

As reported by others,^9,10^ the present study found an elevated rate of infections in patients receiving dialysis via CVCs and observed an infection rate in non-tunneled CVCs approximately five times greater than the rate in tunneled CVCs, which reveals the importance of current recommendations that non-tunneled CVCs should only be temporary, used for less than 2 weeks.^11^

Although other studies have suggested an increased risk of infection in patients with prior hospital admissions,^11^ this was not observed in our sample, probably because of the small number of patients with prior hospital admissions. In the present study, 74.2% of the patients did not have a history of hospital admission within 30 days of enrollment on the study.

Blood cultures are important microbiological tests and can identify the germs causing an infection and guide antimicrobial treatment, by analysis of the antimicrobial susceptibility of the microorganism detected.^6,12^ It is recommended that blood cultures should preferably be performed with paired aerobic and anaerobic flasks.^13^ Use of an aerobic flask only is often the policy at institutions that have problems with reimbursement by insurers and health plans and/or because of logistics issues related to transport/storage of samples before cultures are seeded.^14^ The center analyzed in this study routinely performed aerobic only cultures, primarily because of logistics issues related to the need for transport to an independent laboratory in a short period of time, making it difficult to perform anaerobic cultures, which is a barrier that has been reported in other similar studies.^15^

A range of different initiatives have been studied to reduce the time taken to identify and test the antimicrobial susceptibility of pathogens. All of these methods can be compromised by transport delays or other factors, resulting in delays to incubation and diagnostic yield.^14^ In low and medium income countries, blood culture flasks are probably stored at tropical temperatures, which may exceed the recommended incubation temperatures.^14^ At the center studied in this research, blood culture flasks are transported to a commercial laboratory, which only works during business hours. The flasks are stored in room air termperature and may wait for more than 4 hours before sample processing begins. The elevated number of negative blood cultures found in the present study (52.6%) may be related to the fact that the dialysis center does not have an in-house microbiology service, causing delays with processing of samples, as has also been reported in prior studies.^14,16^

The literature describes a high proportion of gram-positive bacteria as causing these infections, particularly *Staphylococcus aureus,* since skin pathogens are the most common contaminants of CVCs. However, the most frequently isolated microorganism in this study was *Staphylococcus hominis,* which is the third most frequent coagulase-negative staphylococcus species isolated from the blood of hospitalized patients.^17^ It is potentially opportunistic and capable of causing bloodstream infections, particularly in immunocompromised patients, such as chronic kidney patients on hemodialysis. Data from Brasil^18^ show an increase in gram-negative pathogens in confirmed CVC-associated primary bloodstream infections, which we also observed in the present series, with a little over half of the positive blood cultures growing gram-negative bacteria.

In the present study, we identified the type of CVC utilized as a relevant risk factor for infection, with a five times greater infection rate in non-tunneled CVCs. Taylor et al.^19^ studied 11 Canadian dialysis units and also found a significantly lower risk of infection with tunneled CVCs compared with non-tunneled CVCs.

With regard to the number of hemodialysis sessions per week, we observed that patients who had dialysis twice per week had a higher rate of infection than patients receiving dialysis three times per week. The effect of dialysis dosage in the context of bacteremia is uncertain. Observational studies report conflicting data, some suggesting that a higher dose of dialysis could reduce the incidence of infections in chronic renal patients on dialysis.^20,21^ However, a reanalysis of the large HEMO randomized study,^22^ with 15 participating dialysis centers and 1,846 patients, did not detect a reduction in infection rates among patients on higher doses of dialysis.^21,22^

With regard to infection rates, in the present study we observed 10.1 events per 1,000 catheter days, at rates of 15.1 in non-tunneled catheters and 3.3 in tunneled catheters. Comparing this with published data,^6,7^ the incidence of CVC-related bacteremia varies from 1.1 to 5.5 episodes/1,000 catheter days. We believe that the high number of patients undergoing dialysis via non-tunneled catheters is responsible for the greater-than-average overall infection rate. The frequency of infections in tunneled CVCs only (3.3 events/1,000 catheter days) was within the range described in the literature.^6,7,23^

In the current Brazilian dialysis scenario, we see a continuous increase in the proportion of patients with tunneled CVCs, in an attempt to reduce infectious foci, when compared to the quality of access via non-tunneled CVCs, although the ideal prevention would be large-scale substitution of CVCs, whether tunneled or not, with AVFs.^6^ In Brazil, with the exception of some specific regions, there are difficulties with access to vascular surgeons to create AVFs, leading to increasing numbers of patients with failed vascular access and/or contingency vascular accesses, because of central venous lesions caused by use of multiple previous CVCs.^24^ This problem is evident in our series, in which less than 1/5 of the patients had their CVCs removed to start using a mature AVF.

The present study presents limitations directly related to difficulties with data collection, since we were faced with: (1) occasional difficulties with collecting data directly from patients during hemodialysis sessions; (2) incomplete data in patients’ medical charts; (3) a low number of blood cultures; and (4) a high number of negative blood cultures. Notwithstanding, since this is an observational study, it is nevertheless capable to point ways to improve the care delivered to patients at the dialysis center studied.

## CONCLUSIONS

We conclude that the overall incidence rate of CVC infections was 10.1 events per 1,000 catheter days, breaking down as 15.1 in non-tunneled catheters and 3.3 in tunneled catheters. The predictive factors of infection development identified were use of non-tunneled CVC and two hemodialysis sessions per week. With regard to the microbiological profile, a little over half of the bacteria isolated were gram-negative ones.

## References

[1] Rayner HC, Pisoni RL (2010). The increasing use of hemodialysis catheters: evidence from the DOPPS on its significance and ways to reverse it. Semin Dial.

[2] Hoen B, Paul-Dauphin A, Hestin D, Kessler M (1998). EPIBACDIAL: a multicenter prospective study of risk factors for bacteremia in chronic hemodialysis patients. J Am Soc Nephrol.

[3] Astor BC, Eustace JA, Powe NR (2005). Type of vascular access and survival among incident hemodialysis patients: the Choices for Healthy Outcomes in Caring for ESRD (CHOICE) study. J Am Soc Nephrol.

[4] O’Grady NP, Alexander M, Burns LA (2011). Guidelines for the prevention of intravascular catheter-related infections. Clin Infect Dis.

[5] Mermel LA, Allon M, Bouza E (2009). Clinical practice guidelines for the diagnosis and management of intravascular catheter-related infection: 2009 Update by the Infectious Diseases Society of America. Clin Infect Dis.

[6] Alhazmi SM, Noor SO, Alshamrani MM, Farahat FM (2019). Bloodstream infection at hemodialysis facilities in Jeddah: a medical record review. Ann Saudi Med.

[7] Lafrance JP, Rahme E, Lelorier J, Iqbal S (2008). Vascular access-related infections: definitions, incidence rates, and risk factors. Am J Kidney Dis.

[8] Abramson JH (2011). WINPEPI updated: computer programs for epidemiologists, and their teaching potential. Epidemiol Perspect Innov.

[9] Fram D, Okuno MF, Taminato M (2015). Risk factors for bloodstream infection in patients at a Brazilian hemodialysis center: a case-control study. BMC Infect Dis.

[10] Gauna TT, Oshiro E, Luzio YC, Paniago AM, Pontes ER, Chang MR (2013). Bloodstream infection in patients with end-stage renal disease in a teaching hospital in central-western Brazil. Rev Soc Bras Med Trop.

[11] Lok CE, Huber TS, Lee T (2020). KDOQI Clinical Practice Guideline for Vascular Access: 2019 Update. Am J Kidney Dis.

[12] Ling CL, Roberts T, Soeng S (2021). Impact of delays to incubation and storage temperature on blood culture results: a multi-centre study. BMC Infect Dis.

[13] Araujo MRE (2012). Hemocultura: recomendações de coleta, processamento e interpretação dos resultados. J Infect Control..

[14] Levinson W, Levinson W (2010). Microbiologia médica e imunologia.

[15] Palmer HR, Palavecino EL, Johnson JW, Ohl CA, Williamson JC (2013). Clinical and microbiological implications of time-to-positivity of blood cultures in patients with Gram-negative bacilli bacteremia. Eur J Clin Microbiol Infect Dis.

[16] Melo GB, Melo MC, Carvalho KS, Gontijo PP (2009). *Staphylococcus aureus* e estafilococos coagulase negativos resistentes à vancomicina em um Hospital Universitário Brasileiro. Rev Cienc Farm Basica Apl.

[17] Esmanhoto CG, Taminato M, Fram DS, Belasco AGS, Barbosa DA (2013). Microrganismos isolados de pacientes em hemodiálise por cateter venoso central e evolução clínica relacionada. Acta Paul Enferm.

[18] Brasil (2016). Boletim de Segurança do Paciente e Qualidade em Serviços de Saúde no 14: Avaliação dos indicadores nacionais das Infecções Relacionadas à Assistência à Saúde (IRAS) e Resistência microbiana do ano de 2015.

[19] Taylor G, Gravel D, Johnston L (2002). Prospective surveillance for primary bloodstream infections occurring in Canadian hemodialysis units. Infect Control Hosp Epidemiol.

[20] Wolfe RA, Ashby VB, Daugirdas JT, Agodoa LY, Jones CA, Port FK (2000). Body size, dose of hemodialysis, and mortality. Am J Kidney Dis.

[21] Port FK, Ashby VB, Dhingra RK, Roys EC, Wolfe RA (2002). Dialysis dose and body mass index are strongly associated with survival in hemodialysis patients. J Am Soc Nephrol.

[22] Allon M, Depner TA, Radeva M (2003). Impact of dialysis dose and membrane on infection-related hospitalization and death: results of the HEMO study. J Am Soc Nephrol.

[23] Bevilacqua JL, Gomes JG, Santos VF, Canziani ME (2011). Comparison of trisodium citrate and heparin as catheter-locking solution in hemodialysis patients. J Bras Nefrol.

[24] Neves PDMM, Sesso RCC, Thomé FS, Lugon JR, Nasicmento MM (2020). Brazilian Dialysis Census: analysis of data from the 2009-2018 decade. J Bras Nefrol.

