# Supplementary material for: Predictive factors of infection in patients with chronic kidney disease using hemodialysis catheters
Source: J Vasc Bras. 2023 Aug 11;22:e20220098. doi: 10.1590/1677-5449.202200982 (PMC10545231; doi:10.1590/1677-5449.202200982)
Supplement: Translation

# Translation: Fatores preditivos de infecção em pacientes renais crônicos em uso de cateteres venosos centrais

**DOI:** 10.1590/1677-5449.202200981

**Authors:** Juliana da Costa Matos, Laura Lane Menezes Polsin, Karla Cristina Petrucelli Israel, Leonardo Pessoa Cavalcante

**Affiliations:** 1 Universidade Federal do Amazonas - UFAM, Manaus, AM, Brasil.; 2 Universidade do Estado do Amazonas - UEA, Manaus, AM, Brasil.

**Keywords:** diálise renal, unidades hospitalares de hemodiálise, infecções relacionadas a cateter, cateteres de demora

## Abstract

**Contexto:**

Infecção é a complicação mais frequente do uso de cateter venoso central em hemodiálise.

**Objetivo:**

O propósito do trabalho foi determinar a taxa de incidência de infecções de cateteres venosos centrais para hemodiálise em um centro de diálise no estado do Amazonas, bem como seus fatores preditivos, além de traçar o perfil microbiológico dessas infecções.

**Métodos:**

Trata-se de um estudo observacional, com dados coletados mensalmente e de forma prospectiva, por meio de entrevista e análise de prontuários de pacientes submetidos a hemodiálise por meio de cateteres venosos centrais em um centro de diálise durante um período de 12 meses.

**Resultados:**

Foram analisados 96 cateteres venosos centrais, de 48 pacientes. Do total, foram 78 cateteres venosos não tunelizados (81,3%) e 18 cateteres venosos tunelizados (18,7%). Dos cateteres acompanhados, 53,1% foram trocados por motivo de infecção, sendo realizada hemocultura de 35,2% dos pacientes que apresentaram infecção de cateter. Quanto ao perfil microbiológico, das nove hemoculturas positivas, em cinco foram isoladas bactérias gram-negativas, e em quatro foram isoladas bactérias gram-positivas. A bactéria mais frequentemente isolada foi a *Staphylococcus hominis*, presente em 22,2% das hemoculturas positivas.

**Conclusão:**

A taxa de incidência global de infecção de cateteres venosos centrais foi de 10,1 episódios por 1.000 dias de cateter, sendo de 15,1 nos cateteres não tunelizados e de 3,3 nos cateteres tunelizados. Os fatores preditivos identificados foram o uso de cateter venoso central não tunelizado e a realização de duas sessões de diálise semanais. Quanto ao perfil microbiológico, pouco mais da metade das bactérias isoladas foram gram-negativas.

## INTRODUÇÃO

O tratamento hemodialítico é feito por meio de um acesso vascular de curta ou longa permanência. Para o acesso de curta permanência, utilizam-se os cateteres venosos centrais (CVCs) não tunelizados (sem *cuff* subcutâneo), e para o acesso de longa permanência, são preconizados os CVCs tunelizados (com *cuff* subcutâneo) ou as fístulas arteriovenosas (FAVs), como acessos definitivos. Os CVCs constituem o acesso vascular inicial mais utilizado para hemodiálise, mesmo em pacientes que eram acompanhados por nefrologistas até 4 meses antes do início da terapia renal substitutiva (TRS)^1^ . A complicação mais frequente relacionada ao uso dos CVCs para hemodiálise é a infecção, sendo a simples presença do CVC o principal fator de risco para bacteremia/infecção^2^ . Astor et al.^3^ constaram um risco 47% maior de mortalidade em pacientes que utilizam CVCs para hemodiálise, em comparação a pacientes que dialisavam por meio de FAVs.

A infecção dos CVCs manifesta-se, clinicamente, por febre e/ou calafrios, além de eventuais sinais locais de infecção, como hiperemia e/ou secreção no local de inserção do cateter ou próximo ao mesmo^4^ . O diagnóstico etiológico da infecção relacionada ao cateter, segundo a Infectious Disease Society of America^5^ , é realizado pelo crescimento do mesmo microrganismo em uma amostra de sangue periférico e em uma amostra da ponta do cateter ou em duas amostras de sangue periférico distintas.

Frente a esse cenário, o presente trabalho buscou determinar a taxa de incidência de infecção relacionada aos CVCs para hemodiálise e seus possíveis fatores preditivos, bem como traçar o perfil microbiológico dessas infecções em um centro de diálise no Amazonas.

## MÉTODOS

Trata-se de um estudo observacional prospectivo de incidência, aprovado pelo Comitê de Ética em Pesquisa da Universidade Federal do Amazonas (parecer número 4.231.319) e conduzido entre outubro de 2020 e outubro de 2021 no Centro de Doenças Renais do Amazonas, um centro privado especializado no tratamento de pacientes portadores de doenças renais, conveniado ao Sistema Único de Saúde e que apresenta, em média, 240 pacientes em programa de hemodiálise.

A amostra foi composta por pacientes portadores de doença renal crônica (DRC), em programa de hemodiálise, submetidos a três sessões de hemodiálise por semana, com duração média de 3 horas e meia e em uso de CVCs. Os critérios de inclusão foram: 1) ser paciente renal crônico; e 2) estar dialisando por meio de CVC implantado no centro de diálise. O critério de exclusão foi apresentar outro foco infeccioso. Quanto ao cálculo do tamanho da amostra, considerando que 54 pacientes dialisavam por meio de cateteres venosos centrais (Figura 1), utilizando um nível de confiança de 95%, um erro amostral de 5%, e assumida uma proporção de 55% de frequência de bacteremia em cateteres venosos centrais^6,7^ , o número mínimo de 48 pacientes foi considerado necessário para compor a amostra. Para o cálculo amostral, foi utilizado o software WinPEPI^8^ .

**Figura 1:** Fluxograma da elegibilidade da amostra de cateteres venosos centrais no Centro de Doenças Renais do Amazonas.

Os dados foram colhidos por meio de entrevista direta com os pacientes durante as sessões de hemodiálise e também por meio da coleta de informações adicionais em seus prontuários. Foi utilizado um formulário estruturado que levantou os dados demográficos, exames laboratoriais, comorbidades, detalhes da TRS atual do paciente, sorologia, história pregressa de intervenções e hospitalizações, tempo de DRC, sinais e sintomas apresentados pelo participante, se houve diagnóstico de infecção sistêmica e/ou infecção no local de inserção do cateter, resultado da hemocultura, tratamento empírico utilizado e desfecho do quadro infeccioso.

Os dados foram armazenados em planilha Excel e analisados com o software *Statistical Package for the Social Sciences* - SPSS, versão 21.0. A normalidade da distribuição dos dados quantitativos foi verificada com o teste Kolmogorov Smirnov, com as variáveis com distribuição normal sendo descritas na forma de média e desvio padrão e, aquelas com distribuição assimétrica, por mediana e intervalo interquartil. As variáveis categóricas foram apresentadas em valores absolutos e relativos. A comparação entre as variáveis quantitativas foi avaliada com os testes *t* de Student e Mann-Whitney e, a associação entre as variáveis categóricas, com os testes qui-quadrado de Pearson e exato de Fischer. Foram consideradas estatisticamente significativas as análises com p < 0,05.

## RESULTADOS

Foram avaliados 48 pacientes (Figura 1) com média de idade de 54,3±14,4 anos, intervalo de 24 e 83 anos (p = 0,909), em maior número do gênero feminino (52,1%) (p =0,554), portadores de diabetes mellitus (DM) (60,4%) (p = 0,420) e de hipertensão arterial sistêmica (HAS) (89,4%) (p = 0,504). Mais frequentemente, eram eutróficos (37,0%) ou tinham sobrepeso (39,1%) (p = 0,565). A maioria dos pacientes era submetida à diálise três vezes por semana (91,7%) (p = 0,009), não tinha sido internada nos últimos 30 dias (74,2%) (p = 0,490) e não era transplantada (94,3%) (p = 0,369). Entre os cinco pacientes transplantados, dois tinham sido operados há 10 anos e outros dois há 21 anos (Tabela 1) (p = 0,999).

**Tabela 1:** Infecção associada às características sociodemográficas, antropométricas, clínicas e de saúde de pacientes portadores de doença renal crônica em programa de hemodiálise no Centro de Doenças Renais do Amazonas, considerados todos os atendimentos (n = 96).

**Características**	**Infeção**		**p**
	**Não**	**Sim**	
	**n = 42 n (%)**	**n = 54 n (%)**	
Sociodemográficas			
Idade em anos (média±DP)	54,7±14,2	55,1±12,9	0,909^a^
Gênero			0,554^b^
Masculino	20 (47,6)	29 (53,7)	
Feminino	22 (54,4)	25 (46,3)	
Antropométricas			
Peso em kg (média±DP)	68,8±12,9	67,2±11,4	0,527^a^
Altura em m (média±DP)	1,6±0,1	1,6±0,1	0,246^a^
IMC em kg/m^2^ (média±DP)	25,7±4,6	25,8±3,8	0,881^a^
Classificação estado nutricional			0,565^c^
Magreza (< 18,5)	4 (9,8)	2 (3,9)	
Eutrofia (18,5-24,9)	14 (34,1)	24 (47,1)	
Sobrepeso (25-29,9)	11 (26,8)	13 (25,5)	
Obesidade (30-39,9)	12 (29,3)	12 (23,5)	
Análises laboratoriais			
Hemoglobina em g/dL (média±DP)	9,6±1,8	9,4±1,4	0,467^a^
Albumina em g/L (média±DP)	3,7±05	4,0±1,4	0,233^a^
Ferro em µg/dL (mediana e II)	49 (38-70)	57 (43-78)	0,279^d^
Ferritina em ng/mL (mediana e II)	228 (124-724)	500 (192-670)	0,306^d^
Doenças preexistentes			
Diabetes mellitus			0,420^b^
Não	16 (38,1)	25 (46,3)	
Sim	26 (61,9)	29 (53,7)	
Tempo diagnóstico de DM em anos	20 (13-22)	17 (15-23)	0,943^d^
Hipertensão arterial sistêmica			0,504^c^
Não	3 (7,1)	7 (13,2)	
Sim	39 (92,9)	46 (86,8)	
Tempo diagnóstico HAS em anos (mediana e II)	7 (3-10)	7 (3-18)	0,902^d^
Periodicidade semanal de diálise			0,009^a^
Duas vezes	0 (0,0)	8 (14,8)	
Três vezes	42 (100,0)	46 (85,2)	
Internação nos últimos 30 dias			0,490^b^
Não	22 (78,6)	27 (71,1)	
Sim	6 (21,4)	11 (28,9)	
Transplantado			0,369^a^
Não	39 (97,5)	43 (91,5)	
Sim	1 (2,5)	4 (8,5)	
Transplantado há quantos anos			0,999^a^
10	1 (100,0)	1 (25,0)	
13	0 (0,0)	1 (25,0)	
21	0 (0,0)	2 (50,0)	

a Teste *t* de Student.

b Teste qui-quadrado de Pearson.

c Teste exato de Fischer.

d Teste de Mann-Whitney.

DP = desvio padrão; DM = diabetes mellitus; HAS = hipertensão arterial sistêmica; IMC = índice de massa corporal; II = Intervalo interquartil.

Nota: os dados faltantes foram um para hipertensão arterial sistêmica, altura, índice de massa corporal e classificação estado nutricional; 15 para tempo de diagnóstico de diabetes mellitus; 20 para hemoglobina; 32 para albumina e ferro; 35 para ferritina; e 43 para tempo de diagnóstico de hipertensão arterial sistêmica.

Os pacientes foram avaliados em consultas no Centro de Doenças Renais do Amazonas, e a frequência de pacientes em relação ao número de trocas de cateter está representada na Figura 2. Um maior número de pacientes fez uma (39,6%) ou duas trocas de cateter (41,7%). Foi acompanhado o desempenho de 96 cateteres durante o período da pesquisa e, considerando o total de avaliações, a maioria dos pacientes utilizava, previamente, cateter não tunelizado (81,7%) (p = 0,270) e teve o mesmo tipo de cateter reimplantado no momento da pesquisa (81,3%) (p = 0,100). Mais frequentemente, o local do acesso era a veia jugular direita (33,3%) (p = 0,382), e a maioria teve a infecção como motivo de troca (53,1%) (p = 0,011). Um maior número apresentou sorologia negativa (94,8%) (p = 0,129) e bacteremia positiva (64,9%) (p = 0,388) com uma mediana de dois episódios de bacteremia ao longo do acompanhamento (p = 0,120). Em relação às trocas, a maioria necessitou trocar o local prévio do acesso (73,4%) (p = 0,936) (Tabela 2).

**Figura 2:** Frequência de pacientes portadores de doença renal crônica em programa de hemodiálise no Centro de Doenças Renais do Amazonas, de acordo com o número de trocas de cateter (n = 48).

**Tabela 2:** Frequência de variáveis relacionadas à diálise, ao acesso e à análise e identificação de infeção do acesso de pacientes portadores de doença renal crônica em programa de hemodiálise no Centro de Doenças Renais do Amazonas (n = 96).

**Variáveis**	**n (%)**
Acesso em uso, prévio à pesquisa	
Cateter tunelizado	5 (5,4)
Cateter não tunelizado	76 (81,7)
Fístula arteriovenosa	7 (7,5)
Nenhum	5 (5,4)
Acesso implantado no momento da pesquisa	
Cateter tunelizado	18 (18,8)
Cateter não tunelizado	78 (81,3)
Local do acesso	
Femoral direita	19 (19,8)
Femoral esquerda	16 (16,7)
Jugular direita	32 (33,3)
Jugular esquerda	21 (21,9)
Veia subclávia direita	4 (4,2)
Veia subclávia esquerda	4 (4,2)
Motivo da troca do acesso no momento da pesquisa	
Infecção	51 (53,1)
Acesso tunelizado	10 (12,0)
Sangramento	2 (2,4)
Sem fluxo	16 (19,3)
Trombose	4 (4,8)
Sorologia	
Negativa	91 (94,8)
Hepatite B	1 (1,0)
Hepatite C	4 (4,2)
Bacteremia	
Não	26 (35,1)
Sim	48 (64,9)
Número de episódios de bacteremia (mediana e II)	2 (1-3)
Internação nos últimos 30 dias	
Não	49 (74,2)
Sim	17 (25,8)
Houve troca de localização prévia do acesso	
Não	25 (26,6)
Sim	69 (73,4)
Utilização de fio-guia para inserção do novo cateter	
Não	24 (25,5)
Sim	70 (72,9)
Hemocultura	
Não	75 (78,9)
Sim	20 (21,1)
Número de amostra para hemocultura	
Uma	1 (11,1)
Duas	8 (88,9)
Amostras não coletadas na ponta do cateter	95 (100,0)
Número de dias livres de infecção (mediana e II)	39,5 (17,0-75,0)

II = intervalo interquartil.Os dados faltantes foram: um para hemocultura; dois para houve troca de localização prévia do acesso e utilização de fio-guia para inserção do novo cateter; três para acesso prévio à pesquisa; 13 para motivo da troca do acesso no momento da pesquisa; 22 para bacteremia; 30 para internações nos últimos 30 dias.

No grupo avaliado, 54 cateteres apresentaram infecção durante o período da pesquisa (56,3%), ocorrência mais frequente entre os desfechos avaliados (Figura 3). Ao todo, 80% dos CVCs não tunelizados infectaram durante o período da pesquisa e apenas 13% dos CVCs tunelizados infectaram durante esse período (p = 0,100). Somente 17,7% dos CVCs foram retirados em decorrência do início de utilização de uma FAV madura. Mais frequentemente, os pacientes com infeção realizavam diálise duas vezes na semana, enquanto aqueles sem infecção realizavam três diálises semanais (p = 0,009). Não foi observada diferença estatisticamente significativa entre as demais características analisadas, segundo a presença ou não de infecção (Tabela 1).

**Figura 3:** Frequência dos desfechos, em todos os atendimentos, relacionados ao acesso de pacientes portadores de doença renal crônica em programa de hemodiálise no Centro de Doenças Renais do Amazonas (n = 96).

Pacientes com infecção tiveram mais frequentemente a trombose como motivo para troca do acesso, enquanto aqueles sem infecção trocaram o acesso pela colocação de cateter tunelizado (p = 0,011). Nos pacientes com infecção, foi realizada hemocultura, enquanto naqueles sem infecção, não (p < 0,001) (Tabela 3).

**Tabela 3:** Infecção associada a variáveis relacionadas à diálise, ao acesso e à análise e identificação de infeção do acesso de pacientes portadores de doença renal crônica em programa de hemodiálise no Centro de Doenças Renais do Amazonas, considerando todos os atendimentos (n = 96).

**Características**	**Infecção**		**p**	
	**Não**	**Sim**		
	**n = 42 n (%)**	**n = 54 n (%)**		
Acesso em uso, prévio à pesquisa			0,270^a^	
Cateter tunelizado	1 (2,4)	4 (7,8)		
Cateter não tunelizado	38 (90,5)	38 (74,5)		
Fístula arteriovenosa	2 (4,8)	5 (9,8)		
Nenhum	1 (2,4)	4 (7,8)		
Acesso implantado no momento da pesquisa			0,100^b^	
Cateter tunelizado	11 (26,2)	7 (13,0)		
Cateter não tunelizado	31 (73,8)	47 (87,0)		
Local do acesso			0,382^a^	
Femoral direita	7 (16,7)	12 (22,2)		
Femoral esquerda	10 (23,8)	6 (11,1)		
Jugular direita	13 (31,0)	19 (35,2)		
Jugular esquerda	7 (16,7)	14 (25,9)		
Veia subclávia direita	2 (4,8)	2 (3,7)		
Veia subclávia esquerda	3 (7,1)	1 (1,9)		
Motivo da troca do acesso durante a pesquisa:			0,011^a^	
Infecção	22 (52,4)	29 (53,7)		
Acesso tunelizado	9 (21,4)	1 (1,9)		
Sangramento	1 (2,4)	1 (1,9)		
Sem fluxo	8 (19,0)	8 (14,8)		
Trombose	0 (0,0)	4 (7,4)		
Motivo da troca não descrito no prontuário e desconhecido pelo paciente	2 (4,8)	11 (20,4)		
Sorologia			0,129^a^
Negativa	42 (100,0)	49 (90,7)	
Hepatite B	0 (0,0)	1 (1,9)	
Hepatite C	0 (0,0)	4 (7,4)	
Bacteremia			0,388^b^
Não	13 (40,6)	13 (31,0)	
Sim	19 (59,4)	29 (69,0)	
Número de episódios de bacteremia (mediana e II)	2,5 (2,0-3,0)	1,5 (1,0-3,0)	0,120^c^
Houve troca de localização prévia do acesso			0,936^b^
Não	11 (26,2)	14 (26,9)	
Sim	31 (73,8)	38 (73,1)	
Utilização de fio-guia para inserção do novo cateter			0,895^b^
Não	11 (26,2)	13 (25,0)	
Sim	31 (73,8)	39 (75,0)	
Hemocultura			< 0,001^b^
Não	40 (97,6)	35 (64,8)	
Sim	1 (2,4)	19 (35,2)	
Número de amostra para hemocultura			0,999^a^
Uma	0 (0,0)	1 (12,5)	
Duas	1 (100,0)	7 (87,5)	
Número de dias livres de infecção (mediana e II)	39 (19-70)	40 (16-85)	0,960^c^

a Teste exato de Fischer.

b Teste qui-quarado de Pearson.

c Teste Mann-Whitney. II = intervalo interquartil.

Os dados faltantes foram: um para hemocultura; dois para houve troca de localização prévia do acesso e utilização de fio-guia para inserção do novo cateter; três para acesso prévio à pesquisa; 22 para bacteremia; 30 para internações nos últimos 30 dias.

Foi realizada a hemocultura em 19 (35,2%) (p < 0,01) pacientes que apresentaram infecção de cateter, mais frequentemente com duas amostras (88,9%) (p = 0,999). Em nove pacientes (47,4%), houve crescimento bacteriano, sendo que em cinco houve o crescimento de bactérias gram-negativas, e em quatro houve o crescimento de bactérias gram-positivas. Apesar de ter havido mais culturas positivas para bactérias gram-negativas, a bactéria mais frequentemente isolada foi a *Staphylococcus hominis,* gram-positiva, isolada em duas das nove hemoculturas positivas (Figura 4).

**Figura 4:** Frequência do resultado das hemoculturas realizadas em todos os atendimentos de pacientes portadores de doença renal crônica em programa de hemodiálise no Centro de Doenças Renais do Amazonas (n = 19).

A incidência de infeção foi superior nos cateteres não tunelizados em relação aos tunelizados (Figura 5). A mediana global de dias livres de infecção foi de 39,5 dias (17,0-75,0) (Tabela 2).

**Figura 5:** Incidência cumulativa de infecção, de acordo com o tipo de cateter, em pacientes portadores de doença renal crônica em programa de hemodiálise no Centro de Doenças Renais do Amazonas (n = 96). CVC = cateter venoso central.

Na Figura 6, está representada a linha do tempo com a mediana de dias sem infecção entre a data base e a primeira necessidade de troca, e assim sucessivamente. O n corresponde ao número de pacientes em cada troca.

**Figura 6:** Linha do tempo da mediana de dias livres de infecção entre cada necessidade de troca em pacientes portadores de doença renal crônica em programa de hemodiálise no Centro de Doenças Renais do Amazonas.

O número de dias sem infecção apresentou uma mediana de 79 dias [intervalo de confiança de 95% (IC95%) 48-110] (p = 0,960). Do total de pacientes avaliados, 98,9% ficaram 4 dias sem infecção, 51,1% ficaram 78 dias sem infecção e 19,5% ficaram 176 dias sem infecção (Figura 7).

**Figura 7:** Curva de Kaplan-Meier de dias livres de infecção em pacientes portadores de doença renal crônica em programa de hemodiálise no Centro de Doenças Renais do Amazonas (n = 94).

## DISCUSSÃO

A prevenção e o controle de infecções em pacientes renais crônicos em programa de hemodiálise via CVC é uma preocupação constante entre profissionais de saúde. Alhazmi et al.^6^ , em um estudo retrospectivo na Arábia Saudita que analisou 160 pacientes, correlacionaram o uso de CVCs como fator de risco independente para infecção. O tipo de acesso vascular é um fator de risco reconhecido para a infecção de corrente sanguínea entre pacientes em hemodiálise, sendo as FAVs o acesso vascular ideal por proporcionar menores taxas de complicações infecciosas^9^ .

No presente estudo, como já descrito por outros^9,10^ , encontramos uma elevada taxa de infecção em pacientes dialisando por meio de CVCs, sendo a taxa de infecção dos CVCs não tunelizados aproximadamente cinco vezes maior do que a taxa de infecção dos CVCs tunelizados, o que demonstra a importância das recomendações atuais da utilização de CVCs não tunelizados apenas de forma temporária, por um período não superior a 2 semanas^11^ .

A despeito de outros trabalhos sugerirem um risco aumentado de infecção em pacientes com hospitalização prévia^11^ , isso não foi encontrado em nossa amostra, provavelmente pelo número pequeno de pacientes com hospitalização prévia. Na presente investigação, 74,2% dos pacientes não apresentavam histórico de hospitalização nos 30 dias anteriores à sua entrada no estudo.

As hemoculturas são testes microbiológicos importantes, podendo tanto identificar o germe causador da infecção quanto guiar a terapêutica antimicrobiana, por meio da análise da suscetibilidade do germe isolado aos antimicrobianos^6,12,^. Recomenda-se que, preferencialmente, as hemoculturas incluam frascos pareados de hemocultura aeróbia e anaeróbia^13^ . O uso somente de um frasco aeróbio, muitas vezes, é preconizado em instituições em que há dificuldades relativas a acordos de ressarcimento por parte das fontes pagadoras e/ou por questões logísticas relacionadas ao transporte/armazenamento das amostras antes da semeadura^14^ . No centro deste estudo, é realizada rotineiramente a cultura apenas para aeróbios, principalmente por questões logísticas relacionadas à necessidade de transporte em curto período de tempo para laboratório terceirizado, dificultando a realização de culturas para anaeróbios, obstáculo também relatado em outras séries semelhantes^15^ .

Diversas iniciativas foram estudadas para reduzir o tempo para identificar e testar a suscetibilidade antimicrobiana de patógenos. Todas essas tentativas podem ser prejudicadas por atrasos no transporte ou outros fatores, levando a atrasos na incubação e no rendimento do diagnóstico^14^ . Em países de baixa ou média renda, os frascos de hemoculturas são provavelmente armazenados em temperaturas tropicais, podendo exceder as temperaturas de incubação recomendadas^14^ . Dentro do centro estudado na pesquisa, os frascos de hemocultura coletados são transportados para laboratório terceirizado, que funciona em horário comercial. Esses frascos são armazenados em ar ambiente e podem aguardar um período superior a 4 horas até o início do processamento das amostras. O elevado número de hemoculturas negativas encontrado no presente estudo (52,6%) pode estar relacionado com o fato de não existir um serviço de microbiologia inserido dentro do centro de diálise, ocasionando um retardo no processamento das amostras, como também já apontado em outras séries^14,16^ .

A literatura aponta um alto índice de bactérias gram-positivas como causadoras de infecção, principalmente a *Staphylococcus aureus,* uma vez que os patógenos de pele são os maiores contaminantes dos CVCs. Contudo, neste estudo o micro-organismo mais frequentemente isolado foi o *Staphylococcos hominis,* que é a terceira espécie de estafilococos coagulase-negativa mais frequentemente isolada no sangue de pacientes hospitalizados^17^ , sendo potencialmente oportunista, capaz de causar infecções de corrente sanguínea, particularmente em pacientes imunocomprometidos, como renais crônicos em hemodiálise. Dados do Brasil^18^ mostram um aumento dos patógenos gram-negativos em infecções primárias de corrente sanguínea confirmadas associadas a CVCs, o que também encontramos nesta série, com pouco mais da metade das hemoculturas positivas apresentando o crescimento de bactérias gram-negativas.

No presente estudo, encontramos o tipo do CVC utilizado como fator de risco relevante para infecção, tendo sido cinco vezes maior a taxa de infecção em CVCs não tunelizados. Taylor et al.^19^ , estudando 11 unidades de diálise canadenses, encontraram também risco significativamente menor de infecção em CVCs tunelizados, quando comparados a CVCs não tunelizados.

Quanto ao número de sessões de hemodiálise por semana, observamos que os pacientes que dialisavam duas vezes por semana tiveram maior taxa de infecção do que os pacientes que dialisavam três vezes por semana. O efeito na dose de diálise no contexto de bacteremia é incerto. Os estudos observacionais apresentam dados conflitantes, alguns sugerindo que uma maior dose de diálise poderia diminuir a incidência de quadros infecciosos em pacientes renais crônicos dialíticos^20,21^ . Porém, na reanálise do grande estudo randomizado HEMO^22^ , do qual participaram 15 centros de diálise e 1.846 pacientes, não se observou redução na taxa de infecção em pacientes que receberam maior dose de diálise^21,22^ .

Com relação à infecção, encontramos, no presente estudo, 10,1 eventos por 1.000 dias de cateter, sendo de 15,1 nos cateteres não tunelizados e 3,3 nos cateteres tunelizados. Ao comparar com dados de literatura^6,7^ , a incidência de bacteremia relacionada a CVC varia de 1,1 a 5,5 episódios/1.000 cateteres/dia. Acreditamos que o grande número de pacientes dialisando por meio de cateter não tunelizado seja responsável por uma taxa global de infecção acima da média. Considerando apenas a frequência de infecção em CVCs tunelizados (3,3 eventos/1.000 cateteres/dia), estamos dentro do intervalo descrito na literatura^6,7,23^ .

No atual cenário brasileiro de diálise, visualizamos um aumento crescente de pacientes com CVCs tunelizados, na tentativa de diminuição de foco infeccioso, quando comparado à qualidade do acesso por meio de CVCs não tunelizados, sendo que a prevenção ideal seria a substituição em larga escala de CVCs, tunelizados ou não tunelizados, por FAVs^6^ . Em nosso país, com exceção de algumas poucas particularidades regionais, há dificuldade de acesso ao cirurgião vascular para a confecção de FAVs, levando a um aumento de pacientes com falência de acesso vascular e/ou acessos vasculares de exceção, decorrentes de lesões venosas centrais causadas pelo uso de múltiplos CVCs prévios^24^ . Essa dificuldade está evidente em nossa série, em que menos de 1/5 dos pacientes tiveram seus CVCs retirados em decorrência do início da utilização de uma FAV madura.

O presente estudo possui limitações diretamente relacionadas às dificuldades na coleta de dados, uma vez que nos deparamos com: (1) pontuais dificuldades nas informações colhidas diretamente dos pacientes durante as sessões de hemodiálise; (2) dados incompletos nas fichas (prontuários) dos pacientes; (3) um baixo número de hemoculturas coletadas; (4) um elevado número de hemoculturas negativas. Todavia, em se tratando de um estudo observacional, é capaz de apontar caminhos para a melhoria da assistência aos pacientes no centro de diálise estudado.

## CONCLUSÃO

Concluímos que a taxa de incidência global de infecção de CVCs foi de 10,1 eventos por 1.000 dias de cateter, sendo de 15,1 nos cateteres não tunelizados e de 3,3 nos cateteres tunelizados. Os fatores preditivos para o desenvolvimento de infecção identificados foram o uso de CVC não tunelizado e a realização de duas sessões de hemodiálise semanais. Quanto ao perfil microbiológico, pouco mais da metade das bactérias isoladas foram gram-negativas.
